# Advances in SIV/SHIV Non-Human Primate Models of NeuroAIDS

**DOI:** 10.3390/pathogens10081018

**Published:** 2021-08-12

**Authors:** Sonia Moretti, Sara Virtuoso, Leonardo Sernicola, Stefania Farcomeni, Maria Teresa Maggiorella, Alessandra Borsetti

**Affiliations:** National HIV/AIDS Research Center, Istituto Superiore di Sanità, 00162 Rome, Italy; sonia.moretti@iss.it (S.M.); sara.virtuoso@iss.it (S.V.); leonardo.sernicola@iss.it (L.S.); stefania.farcomeni@iss.it (S.F.); mariateresa.maggiorella@iss.it (M.T.M.)

**Keywords:** neuroAIDS, Simian Immunodeficiency Virus, macrophages, macaque, markers, inflammation

## Abstract

Non-human primates (NHPs) are the most relevant model of Acquired Immunodeficiency Syndrome (AIDS) and neuroAIDS, being of great importance in explaining the pathogenesis of HIV-induced nervous system damage. Simian Immunodeficiency Virus (SIV)/ Simian-Human Immunodeficiency Virus (SHIV)-infected monkeys have provided evidence of complex interactions between the virus and host that include host immune response, viral genetic diversity, and genetic susceptibility, which may explain virus-associated central nervous system (CNS) pathology and HIV-associated neurocognitive disorders (HAND). In this article, we review the recent progress contributions obtained using monkey models of HIV infection of the CNS, neuropathogenesis and SIV encephalitis (SIVE), with an emphasis on pharmacologic therapies and dependable markers that predict development of CNS AIDS.

## 1. Introduction

**Figure 1:**
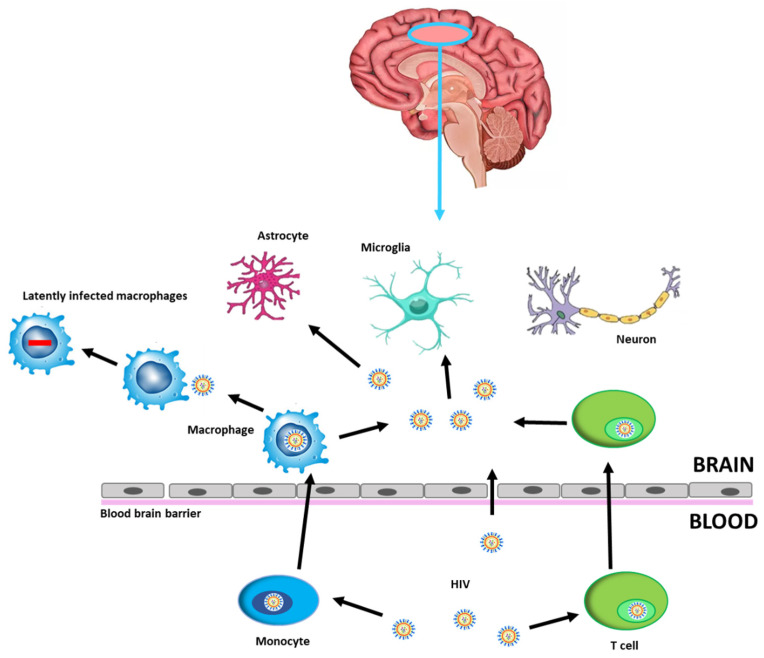
HIV infection into the CNS. HIV can cross the BBB through HIV-1-infected CD4+ T cells or HIV-1-infected monocytes that differentiate into perivascular macrophages, becoming latently infected brain macrophages, or by direct entry, possible in the case of increased permeability. CNS cells susceptible to HIV-1 infection are microglia and astrocytes, while neurons may be damaged by neuroinflammatory processes. Abbreviations: Human Immunodeficiency Virus (HIV), blood–brain barrier (BBB), central nervous system (CNS).

Human Immunodeficiency Virus (HIV)-related cognitive impairments persist with effective antiretroviral therapy (ART) and remain a clinical concern for people with HIV (PWH). Despite use of ART results in effective viral suppression within the systemic circulation, the virus persists in the central nervous system (CNS), as a site of viral reservoir, due to limited ART penetrance, thereby hampering efforts to eradicate HIV. The virus enters the CNS through the blood–brain barrier (BBB) within circulating lymphocytes and macrophages or directly by migrating between microvascular endothelial cells [1,2]. After crossing the BBB, viral particles are able to infect resident cells such as microglia and macrophages thus contributing to establishing a persistent infection (Figure 1).

In the CNS latent integrated HIV DNA can potentially reactivate and replicate viral RNA; alternatively, CNS may be an anatomical site of ongoing, low-level viral replication. Phylogenetic analysis reveals significant compartmentalization of CNS viral populations, since sequences recovered from CSF are phylogenetically distinct from those in blood, showing that CNS may be a source of reactivated viral replication independent from that in the blood. An important unsolved question is whether virus originating in the CNS can reseed the periphery and even cause failure of systemic control in otherwise virologically suppressed individuals, contributing to the development of HIV-associated neurocognitive disorder (HAND) in the course of their life [1,2]. 

Study of mechanisms of neuropathogenesis in the CNS in humans are difficult to assess in vivo. Animal models of CNS HIV infection, such as macaques infected with Simian Immunodeficiency Virus (SIV), offer an ideal opportunity to investigate the immunopathogenesis of HIV-induced CNS damage. Non-human primates (NHPs) have been broadly used for HIV-1/Acquired Immunodeficiency Syndrome (AIDS) pathogenesis and vaccine studies. The infection of macaques closely resembles HIV-1 infection of humans, and together with the use of recombinant Simian-Human Immunodeficiency Virus (SHIV) or HIV-1 derivatives, macaques have become the most commonly used and widely accepted model for HIV/AIDS [3,4,5]. SIV-associated CNS pathology and HAND in macaques are similar to that of HIV-1 in humans [6,7,8]. Characteristics such as lifespan and time to AIDS progression, make the SIV-NHP model suitable for studies of viral latency/reservoir and testing new drugs for neuroAIDS. Over the course of 2–3 years post infection, and in more rapid disease course with neurotropic SIV after about 150 days, approximately 30–40% of infected animals develop AIDS and SIV encephalitis (SIVE), which is the pathological hallmark of neuroAIDS [6,7,8]. The virus enters the CNS as early as 3 days post infection [9,10]. Productive infection is difficult to be revealed during the asymptomatic phase of infection, while following the development of AIDS, the virus is detected in perivascular macrophages, multi-nucleated giant cells (MNGC) and in some cases parenchymal microglia [5,6,7,8,9,10]. Factors that control the development of encephalitis and giant cell formation in the CNS are not well defined, but clearly immune suppression plays an important role [11,12]. The traffic to the CNS of infected macrophage’s precursors is triggered by pro-inflammatory cytokines and chemokines, although microbial translocation together with increased levels of monocyte activation could play an important role [13,14,15]. In the SIV macaque model of AIDS, use of bromodeoxyuridine (BrdU), an analog of thymidine incorporated in DNA during replication, revealed that increased monocyte turnover correlated with the severity of SIVE, confirming the importance of monocyte trafficking [16]. Recent data support the notion that most infected perivascular macrophages and MGNC are present before SIVE lesions are formed, and that local macrophage proliferation, independently of recruitment of monocyte from the periphery, influences lesion formation and inflammation [17,18]. In addition, brain macrophages accumulate virus that reemerges after ART interruption, providing a barrier to HIV eradication [18]. 

## 2. Model of NeuroAIDS and cART

Although various NHP models of disease have provided valuable information on the neuroHIV research, some studies dominate for evaluating the mechanisms of pathogenesis of SIVE/neuroAIDS (Table 1). One model uses immunomodulation by depletion of CD8+ lymphocytes and infection with the viral swarm SIVmac251/239 that results in rapid disease progression and high incidence of SIVE within 3 to 6 months after infection [11,12,19]. This useful and reproducible model offers a rapid progression to neuroAIDS, although a modification of the host immune system, such as depletion of CD8+ T cells, does not effectively reflect the neuropathogenesis of HIV infection of the CNS in humans. Infiltration of CD8+ T cells in the brain plays a key role in the maintenance of viral control in the CNS. CD8+ T cells could act in two opposing ways, protecting from the virus initially and contributing to control of SIV subpopulations and damage during prolonged course of infection [20,21]. The balance between protective and pathologic immune responses in the brain is pivotal, since immune activation has an important role in HAND and other neurodegenerative disorders. In order to induce rapid progression to neuroAIDS, another model employs immune modulation by depletion of CD4+ T lymphocytes prior to SIVmac251 inoculation in rhesus macaques, resulting in productive infection in microglia [22,23]. An additional rapid model of disease and SIVE uses pigtail macaques co-inoculated with immunosuppressive viral swarm SIV/DeltaB670 and the neurovirulent SIVmac/17E-Fr molecular clone that replicates efficiently in macrophages [24,25]. SIV/DeltaB670 induces immunosuppression to allow more efficient replication of SIVmac/17E-Fr, causing rapid disease progression with accompanying CNS disorders, and it has been used to study mechanisms of pathogenesis and innate immunity in the CNS [25]. Regarding the SIVsm804E/rhesus macaque model, virus has been obtained by serially passaged SIVSmE543 in rhesus macaques, resulting in a viral encephalitis model in which the virus replicates efficiently in macrophages, induces neuroAIDS and results in a high incidence of SIVE in a high proportion of rhesus macaques [26]. To develop molecular clones fully representative of the neurovirulent strain, Hirsch and colleagues have realized a neurotropic molecular clone SIVsm804E-CL757 without rapid disease progression and thus more similar to neuroAIDS in HIV-infection [27]. They showed that in macaques that progressed to neuroAIDS, both brain macrophages and memory CD4+T cells harbored replication-competent SIV DNA, however, only brain memory CD4+ T cells harbored SIV DNA in animals without SIVE [27,28]. Recently, Hsu and collaborators have described a new model of SHIV-1157ipd3N4 infection on the CSF and brain parenchyma in rhesus macaques, that mimics the clinical course of early HIV infection in humans more closely than accelerated SIV models. They demonstrated that early neurologic inflammation is related to T cell-mediated inflammation following SHIV infection in the brain and meninges [29].

Historically, the differences in antiretroviral drug (ARV) susceptibility and the lack of an effective ART regimen related to suboptimal pharmacokinetics and the biology of SIVmac infection represented a limitation to study latently infected cells and persistent reservoirs in SIV/SHIV-infected models. Moreover, the BBB contributes to low ART drug penetrance and limited immunosurveillance in the CNS. The improvement of therapeutic regimens over the years, and the achievement of a complete suppression of SIVmac replication in rhesus macaques by a combination of five ARV drugs [30], gave rise to studies of viral persistence, particularly in the CNS. In this context, the SIV model allowed an experimentally controlled infection, the control of ART regimen, and the longitudinally direct evaluations of both RNA and DNA in CSF and brain tissues. However, studies conducted so far demonstrate that, in a SIV macaque model, although ART treatment may suppress virus replication in the periphery and in the brain, viral DNA persists in the latter despite treatment [31]. Another study, performed in accelerated pigtailed macaques SIV model, showed that ART therapy started 12 days after infection did not change SIV DNA levels in the basal ganglia or parietal cortex. This suggests that early treatment does not impact integration of SIV DNA into brain tissue, and thus does not limit formation of potential CNS SIV reservoirs [32]. Another report started ART 4 days post infection in the same animal model, and similarly found no decrease of SIV DNA in brain tissue [33]. Recent studies confirmed the previous results, as untreated and ART-suppressed macaques show comparable frequencies of cells harboring SIVmac251RNA or DNA in brain tissue [34,35]. Similar findings are described also by comparing viremic and ART-suppressed orally-infected infant rhesus macaques [36]. All these studies suggest that virus may enter the CNS early in the course of infection, rapidly integrate as viral DNA, and persist despite ART.

## 3. Virus Persistence in the CNS

The NHP model has been invaluable for studying the persistence of the HIV monocyte/macrophage reservoir under cART, to activate latent reservoirs in vivo, and for detecting replication-competent virus in myeloid cells from the brain [37,38,39,40]. Low turnover rate and resistance to viral cytotoxic effects enable macrophages, specifically microglia and perivascular macrophages, to harbor virus for long periods of time [41] and to release abundant viral products, providing a mechanism for continuous inflammation and production of significant clinically relevant pathology within the CNS. Resident tissue macrophages infected with SIV have the potential to divide and expand viral reservoirs in tissues [42]. By quantitative viral outgrowth assay (M-QVOA) it has been shown that the number of productively infected macrophages in a given tissue, including the brain, is similar from macaque to macaque, whereas the number of productively infected macrophages in different tissues from the same SIV-infected macaque varies widely. This suggests a role for the tissue microenvironments in mediating virus infection of macrophages [37,38]. Brain macrophages of ART-suppressed SIV-infected macaques harbor latent viral genomes that can be reactivated during treatment with the protein kinase C (PKC) agonist ingenol-B and the histone deacetylase (HDAC) inhibitor vorinostat, and that are infectious in peripheral blood mononuclear cells (PBMCs) [43]. This suggests that, although macrophages do not release virus as robustly as CD4+ T cells, they are still capable of re-establishing infection after ART interruption [39,40]. Interestingly, different brain regions display focal viral reactivation, as if SIV RNA levels in other parts of the brain (i.e., basal ganglia and parietal cortex) are similar to those detected in the latency reversing agents (LRA) in untreated animals. In another study that used a larger number of SIV-infected macaques treated with ART, latently infected macrophages containing fewer copies of SIV DNA have been identified in 85.7% of ART-suppressed macaques [38]. This study corroborates previous findings that showed the presence of replication-competent SIV in brain macrophages of ART-suppressed macaques, suggesting that brain macrophages may provide a barrier to HIV eradication. Since most current studies in humans only measure rebound of virus in plasma and not in CSF, these studies are likely to overlook a source of virus that significantly contributes to treatment outcomes, potentially leading to additional CNS damage and reinfection of cells both in the brain and in peripheral blood.

## 4. Viral Sequence Evolution and Host Genetics 

The CNS/CSF is an anatomical site where the restriction of HIV viral quasispecies between cells or anatomical sites, defined as compartment, enables viral evolution and divergence from the virus circulating in the peripheral blood [44,45].

Viral evolution gives rise to variants, possibly derived early in infection from founder HIV-1 quasispecies, that lead to the establishment of localized viral subpopulations. Alternately, viral variants may derive from multiple introductions over time of distinct viral subpopulations that have specific viral genotypic/phenotypic features, such as the enhanced capacity to enter cells expressing low levels of CD4, that are macrophages and microglia [46,47]. Individuals with HAND show genetically distinct HIV-1 variants within the CNS that are not detected in the blood, suggesting that isolate viral replication is occurring in the CNS of HIV-1-infected subjects with severe neurological disorders [47,48]. In particular, the evolution of genetically isolated viral populations in the CNS may be linked with the development of the neurological complications of HIV infection and poor prognosis in patients with AIDS [46,47]. Extensive evolutionary analysis is needed to implicate viral evolution as a major proponent of HAND progression, however the ethical limitation of HIV-1-CNS studies in humans renders this type of analysis difficult. Non-human primates provide a powerful model for the study of HIV intra-host evolution and neuropathogenesis because they facilitate extensive tissue collection and detailed analyses of viral populations in blood and tissues. Yen et al. consistently point to early seeding of the CNS within days of infection. They identify macrophage-tropic SIV Env variants from early stage infection of rhesus macaques, closely related to CNS sequences from animals with SIVE. They define novel determinants of SIV macrophage tropism and neutralization sensitivity in two N-linked glycosylation sites in the V2 and C5 regions (N173 and N481), suggesting that macrophage-tropic SIV capable of establishing viral reservoirs in the brain can be present in blood very early in infection [48]. Similarly, data obtained in SIV-infected pigtailed macaques indicate that viral variants detected early in infection in CSF reemerge after ART interruption, implying that these variants are archived in latent proviral DNA, and that the CNS may serve as a viral reservoir [49,50]. Since brain macrophages harbor replication-competent virus capable of re-establishing infection upon treatment interruption [50] it is possible that these cells are responsible for production of compartmentalized viral variants found in this site. In contrast, Rife et al. characterized viral sequences derived from distinct CNS regions in rhesus macaques inoculated with SIVmac251 viral swarm, and observed that sequence compartmentalization occurs solely within brains of macaques with SIVE and it results from late viral entry of virus evolved in periphery and adapted for CNS [51]. They concluded that, despite multiple entry of virus into the brain over the course of infection, in macaques with SIV-associated CNS neuropathology, brain sequence compartmentalization is likely the result of late entry of virus that has acquired through evolution in the periphery sufficient adaptation for the distinct microenvironment of the CNS. Another study by the same authors, using SIVmac251 in naturally progressing rhesus monkeys, identifies similar brain *nef* gene sequences in different macaques indicating convergent evolution. This implicates the presence of strong selection pressures on *nef* gene that generates viral variants may be required for SIV to efficiently invade the CNS and suggests a significant role for *nef* in establishing neurotropic strains [52]. In an elegant study, Matsuda et al. demonstrated compartmentalization of virus populations between the CNS and the periphery by co-inoculation of rhesus macaques with the non-neurovirulent SIVsm-E543-3 and the SIVsm804E-CL757 that targets the CNS. Molecular analysis of tissues from infected macaques revealed that SIVsm804E-CL757 exclusively targeted the CNS whereas SIVsm-E543-3 variants clustered in the periphery, consistent with a role for viral determinants in the mechanisms of neuroinvasion [27]. These results confirm previous studies in juvenile pigtailed macaques based on phylogenetic evaluation of a small region of *env* and indicating that the neurovirulent SIVmac/17E-Fr molecular clone co-inoculated with SIV/DeltaB670 swarm specifically targets the CNS [7,42,43]. Moreover, in the same study authors demonstrated that within the brain of virally ART-suppressed animals, latent virus could be reactivated with LRAs, and that the latent viral genomes are genetically distinct from those in plasma [43]. 

Host genetic factors including MHC class I alleles have been shown to significantly influence the outcome of HIV/SIV infection in their respective hosts [53,54,55,56,57,58] and to play neuroprotective roles in lentiviral-induced CNS disease [58,59,60]. In rhesus macaques, the MHC class I alleles Mamu-A*01 and -B*17 have been correlated with control of systemic viral suppression levels and decreased the rate of disease progression and the development of SIVE [54,55,61]. The effects of MHC class I allele expression on the development of CNS disease in pigtailed macaques have been investigated by Mankowski and collaborators. They showed that the MHC class I allele Mane-A1*084 is neuroprotective; pigtailed macaques that lack this allele are 2.5 times more likely to develop SIVE and have significantly higher CNS SIV replication [50,59]. Allele Mane-A1*084, that presents the prototypic immunodominant SIV Gag epitope termed KP9, also induces K165R escape mutant in this epitope, as shown by the presence of K165R mutation in latent proviral DNA reservoirs including the CNS. Starting from this observation, Beck et al. demonstrated that viral fitness in the CNS is distinct from the periphery. Molecular analysis of viral variants from Mane-A1*084:01-positive macaques, infected with a SIV/17E-Fr, that bears K165R mutation, shows that K165R is stable in plasma and reverts to wild type only in both CSF and in microglia cultures. This result is consistent with decreased fitness of SIV/17E-Fr bearing K165R mutation in CNS. Moreover, Mane-A1*084:01-positive macaques, first vaccinated with a virus-like particle-based Gag KP9 peptide to induce CTL responses on Gag KP9 and then challenged with wild type SIV/17E-Fr, had lower viral load in CSF compared to unvaccinated controls, but showed no difference in plasma viral loads. This result suggests that Mane-A1*084 -associated CTL control is crucial to control viral replication in the CNS [49].

## 5. Neurotoxicity of Viral Proteins

Tat and Nef viral proteins may exert their neurodegenerative actions in the CNS of HIV-infected individuals [62,63]. In tissues of macaques, a wild type SIV molecular clone was found in CSF and brain regions as compared to the *nef*/LTR-deleted SIV molecular clone that was present only in the periphery, suggesting a diminished capability to enter the CNS and decreased neurotropism in the *nef*/LTR-deleted viruses [64]. Lehman and collaborators suggested a model based on Nef-induced CCL2 expression to HIV/SIV neurovirulence [65]. The transfer of Nef by microvesicles into endothelial cells and the subsequent induction of CCL2 stimulated CCR2 signaling in neurons leading to their dysfunction and death [65]. Recently, in a SIV/macaque model it was found that, despite ART therapy, Nef detection is not limited to the cells actively replicating the virus but is also present in neurons; its presence is associated with apoptosis, making Nef a possible contributor of neurological impairment in infected individuals [66]. In a SIV/SHIV macaque model of infection and HAND a relationship was demonstrated between Tat and menin, a tumor suppressor protein required for HIV-1 Tat transactivation [67]. It was found that Tat-induced apoptosis is menin dependent, and the aberrant overexpression of menin induces neuronal apoptosis, indicating that targeting menin may block the Tat transactivation-associated neuronal damages in HAND [67]. The role of Tat in triggering microglial inflammation mediated by a Nod-Like Receptor (NLR) family pyrin domain containing 3 (NLRP3) inflammasomes is reported by in vitro findings and then confirmed in brain samples from SIV-infected macaques, demonstrating that pharmacological inhibition and genetic silencing of NLRP3 could reduce the neuroinflammation and the development of HAND [68]. In a recent report, Tat-mediated induction of astrocytic amyloidosis has been reported. The authors conducted a study using brain tissues of chronically SIV-infected rhesus macaques that evidenced a significant up-regulation of toxic amyloid proteins in specific brain regions, such as the frontal cortex, parietal cortex, cerebellum and brain stem, and no expression in the occipital cortex and thalamus, suggesting a brain region-specific deposition of toxic amyloid proteins [69].

## 6. Biomarkers Predicting CNS Disease Progression 

Identification of predictive non-invasive reliable biomarkers for risk and development of HIV/neuroAIDS from both blood and CSF is becoming a priority. While hematologic markers have the advantage of relatively easy technical collection, neuroinflammation and neuronal degeneration markers may provide insight into the degree of neuroaxonal damage and immune activation in the CNS. In addition, neuroimaging markers have also been proposed as reliable biomarkers to document changes in the size and structure of the brain in the context of SIV/HIV infection. Neurodegenerative changes in virologically-suppressed cART-treated macaques can allow identification of predictive/prognostic markers associated with risk of developing CNS disease for therapeutic intervention in HIV-infected patients prior to the development of neurologic symptoms. Biomarkers described below in the respective sections are listed in Table 2.

### 6.1. Hematologic Markers

High plasma viral RNA is considered predictive of SIV-associated CNS disease. In a pigtailed macaque model, plasma SIV RNA level is elevated during asymptomatic and terminal phase of infection in animals that progress to develop SIV encephalitis compared to those that do not [24]. 

Data reported from the literature clearly indicate utility of hemoglobin and platelet levels as potential prognostic indicators for CNS disease progression [70,71]. Infected monkeys have greater decreases in hemoglobin from baseline compared to uninfected controls, especially in the asymptomatic and chronic stages of the disease [70]. Moreover, macaques that develop SIV encephalitis have a higher decrease in hemoglobin compared to those who did not develop CNS disease [70]. Regarding platelets, a decline from baseline is considered a predictive circulating hematologic marker of retroviral-associated CNS disease and a prognostic indicator of increased risk for the development of SIV-associated CNS disease [70]. Levels of circulating monocytes could be correlated with the development of brain pathology. Bissel and colleagues found that in SIV-infected pigtailed macaques, development of encephalitis is not predicted by changes in circulating activated monocytes [12]. Similarly, another group showed no difference in total monocyte counts between animals that develop encephalitis and those that do not [70], thus considering circulating monocyte or activated circulating monocyte numbers alone not valuable predictive markers of CNS inflammation [70]. Conversely, by labeling monocytes in the bone marrow with BrdU, which is a marker of monocyte proliferation, another study demonstrated that the percentage of monocytes in blood correlates with the development of AIDS and the severity of CNS histopathology [16]. A marker that correlates with the number of BrdU+ monocytes in these animals is soluble CD163 (sCD163) in plasma [16], and it was shown that significantly higher levels of sCD163 were present in the plasma of SIV-infected macaques that develop encephalitis compared to macaques that do not [70].

### 6.2. CSF Markers

CSF biomarkers are considered predictive and prognostic for SIV-associated neurological disease in CNS. Ina pigtailed macaque model of SIV encephalitis, the increase of pro-inflammatory cytokines in CSF during asymptomatic and chronic stages of infection are highly predictive of the development of CNS disease. Monocyte chemoattractant protein 1 (MCP-1) or CCL2, IL-6, and neopterin found in CSF correlate with monocyte and macrophage inflammation and CNS disease [70]. In particular, in animals that progress to SIV encephalitis, CCL2 and IL-6 levels are significantly higher in the CSF during the asymptomatic and terminal phases of infection as compared to animals that do not develop SIV encephalitis, that instead show an acute spike which then normalizes to the baseline level [32,70]. Notably, in the same animals a substantial increase in CSF neopterin is detected, while only negligible amounts are present in macaques that do not develop SIV encephalitis [70]. Thus, CSF neopterin is considered a very specific biomarker of inflammation in the CNS compared to the periphery. Another marker of CSF neuronal damage is human cartilage glycoprotein 39 (YKL-40) produced by microglia following SIV infection [72,73,74]. YKL-40 is upregulated in the CSF of SIV-infected pigtailed macaques that develop encephalitis and correlates with an increase of viral load in CSF [74], while macaques that do not develop encephalitis maintain consistent baseline levels of CSF YKL-40 throughout the course of infection. Neurofilament light chain (NF-L) drastically increases in CNS during asymptomatic and chronic stages of infection in animals that develop encephalitis, and drops in animals that do not [70], showing increased neuroaxonal damage in animals that develop encephalitis. This suggests that CSF neurofilament light chain may be a valuable predictive biomarker for the development of lentiviral-associated CNS disease. The quinolinic acid/tryptophan (QUIN/TRP) ratio, through activation of the kynurenine pathway (KP), has been shown as a sensitive, early predictive biomarker of inflammatory neurological disease in the SIV model [75]. In fact, in samples of striatum, which is one of the principal components of the basal ganglia, the QUIN/TRP ratio strongly correlates with markers of macrophage/microglial activation (CD68, MHC Class II). The striatal QUIN/TRP ratio also correlates significantly with both SIV *gag* RNA and the neuronal damage marker amyloid precursor protein (APP), supporting a direct link between accumulation of neurological damage over time and activation of macrophages/microglia [75]. Moreover, in the cART-treated animals, whose CSF and plasma viral loads had been suppressed, despite no detectable viral RNA in striatum and normalization of CSF metabolite levels, KP metabolites remain elevated in striatum [75]. This suggests that cerebral KP activation is only partially resolved with cART, and that the QUIN/TRP ratio may be considered a predictive biomarker of CNS disease. Interestingly, another study confirmed the elevated levels of Kynurenine (KYN) in the CSF of SIVmac251-infected macaques with high viremia, and assessed inflammatory responses among different anatomical sites in the CNS [76]. The authors found increased expression of proteins involved in inflammation and perivascular leukocyte infiltration in the midbrain, and downregulation of inflammatory proteins as well as lack of perivascular cuffing in the frontal lobe in the absence of differences in SIV transcriptional activity within these tissues [76]. The segregation of inflammatory responses and of different immunologic effects in anatomically separated regions of the CNS is consistent with the hypothesis that specific areas of the CNS may provide an immune-privileged site for SIV/HIV persistence. 

More recently, microRNAs (miRNAs) are considered promising biomarkers for differentiating the stages of progression of HIV-1 infection [70]. In fact, the profile of several miRNAs in plasma are differently expressed in chronically infected individuals and correlate with CD4+ T cell count or with the known time of infection [77,78]. Additionally, some studies suggest that miRNA may play a role in the pathogenesis of HIV-1-induced encephalitis [79,80].

In the SIV monkey model, miR-125b and miR-146a were found to be differentially regulated in both plasma and CSF over the course of infection and disease development [81]. An acute response of miR-125b appears to be more pronounced in CSF than in plasma, while in infected microglial cells increased miR-146, has been found to target CCL8 (or Monocyte Chemoattractant Protein 2 (MCP2)) and to bind to the SIV genome [81]. Yelamanchili and collaborators showed that six miRNAs are significantly upregulated in the caudate, and four in the hippocampus, of monkeys with SIV encephalitis and that three miRNAs (miR-21, miR-142-5p and miR-142-3p) are upregulated in both regions [82,83]. While the potentials of miRNAs as biomarkers for HIV/AIDS disease progression have been demonstrated, there are still some pieces missing from the puzzle before miRNAs can be used for treatment of neuroAIDS as in other CNS viral infections. The drawback with miRNAs is that little is known of their normal function in all HIV target cells, as it appears that each individual cell type has a unique miRNA profile. Overexpression of miRNAs may lead to unintended, off-target and unanticipated side effects. The action of miRNAs remains to be scrutinized before considering these molecules as anti-HIV drugs [84,85].

### 6.3. Omics-Based Analyses 

Significant changes in CSF metabolome may allow to identify specific upregulated metabolites that may have not only neuropathogenic effects, but can also indicate mechanisms of CNS disease progression [86]. Changes in specific metabolite levels in CSF have been associated with SIV infection, for example the increase of concentration of the neurotoxic quinolinic acid, or nitrate and nitrite in CSF [87]. In SIV encephalitis, a mass-based metabolomics study showed elevation in phospholipids and free fatty acids associated with the activation of phospholipases [88]. Proteomic analysis of CSF of SIV-infected monkeys showed increased expression of alpha-1-antitrypsin, complement C3, hemopexin, IgM heavy chain and plasminogen, and the concomitant increased expression of their genes in the brain parenchyma that was linked to CNS disease progression [89].

Although biopsy tissues should not be technically considered as source of biomarkers, omics-based analyses in post-mortem samples of neuroAIDS are worth mentioning. Few studies characterize whole genome transcriptome of protein coding genes in brain tissues of SIV-infected monkeys during acute infection [90,91], or in SIV encephalitis [92]. The frontal lobe of animals with acute infection reveals significantly upregulated expression of 97 genes, of which a large proportion related to interferon and IL-6 [91]. Conversely, in chronically infected animals only seven genes show significant expression changes as compared to uninfected animals, specifically, the interferon-induced genes G1P3 and IFITM1; HLA-A, C and DRα MHC genes; immunoglobulin gene IGHG3; and RANTES. SIV encephalitis is characterized by genes upregulated in the frontal cortex, occipital lobe, midbrain, cerebellum [91], and hippocampus [92], linked to the pathological processes involved in neuroAIDS, including the interferon/STAT1 pathway and monocyte/macrophage migration. Recently, poly (ADP-ribose) polymerase (PARPs) expression in SIV-associated neuropathogenesis has been investigated [93]. Mavian and collaborators reported significant dysregulation of PARP expression in brain tissues with detectable virus, associated with neurodegenerative processes. As already observed in previous studies [93], they found altered transcriptional pathways associated with innate immune response, neuroinflammation, oxidative stress, cellular death, interferon/STAT1 pathway, and monocyte/macrophage migration. Notably, 4 of 18 PARP genes (PARP9, PARP10, PARP12, PARP14) are upregulated, providing evidence that PARPs overexpression may be linked to the presence of virus in the brain [93], and indicating PARP dysregulation as a new, key indicator of SIV brain infection and neuropathogenesis. 

### 6.4. Neuroimaging Markers 

Multiple magnetic resonance imaging (MRI) modalities, including diffusion tensor imaging (DTI), perfusion MRI, in vivo MR spectroscopy (MRS), magnetization transfer ratio (MTR), and positron emission tomography (PET) have demonstrated abnormalities of the SIV-infected CNS. We briefly describe several MRI and PET studies in non-human primate models of neuroAIDS. MRI has been applied in the SIV-infected model to investigate the structural and functional alterations of the brain. MRI is also an ideal model for the longitudinal evaluation of neuro-imaging markers under controlled conditions, especially with the modern high field MRI techniques. A study used DTI and perfusion MRI to access the neurological disease development in SIV-infected macaques and found reduced cerebral blood flow (CBF) and decreased fractional anisotropy (FA) in several regions of interest [94]. In particular, longitudinal changes in CBF of caudate, prefrontal and parietal cortex and in FA of the whole brain, correlate significantly with the CD4+ T cell depletion and with the CD4/CD8 ratio [94]. These findings suggest that quantitative MRI measures could be a biomarker for SIV disease progression, and that CBF and FA may be sensitive image markers to follow the progression of the infection and for characterizing the disease development of HIV infection. Other groups focus on resting-state fMRI (rs-fMRI) in the macaque model as a useful tool to investigate the abnormal spontaneous brain activities caused by SIV infection [95,96]. These studies indicate that some regions, such as the hippocampus, putamen, and frontal and occipital lobe are more vulnerable and correlate with immunological parameters. Furthermore, changes of regional homogeneity (ReHo) are detected in several areas consistent with disperse distribution of astrocytosis [95]. Thus, ReHo value can be utilized as a non-invasive biomarker of spontaneous brain activity changes caused by the progression of neurological impairments. During acute SIV infection, brain injury is shown by significant MTR reduction in the genu, splenium, centrum semiovale, putamen and thalamus [97], and these changes correlate significantly with immune responses. In conclusion, the spatial-temporal MTR changes are closely associated with the progression of SIV infection. 

MRS provides a non-invasive approach to measure cerebral metabolic abnormalities in SIV-infected monkeys. Moreover, the macaque model has similar MR spectroscopic changes as in human neuroAIDS, suggesting that neurochemical and cellular responses to SIV and HIV are similar. In particular, during acute SIV infection, significant reduction of both N-acetylaspartate (NAA)/Creatine (Cr) and choline (Cho)/Cr ratio is observed at 13 and 27 days after inoculation, respectively, and the change of Cho/Cr ratio correlates with plasma viral load [98]. In another study, the Cho/Cr ratio in frontal lobe or NAA/Cr ratio in basal ganglia correlate with plasma viral load [99]. In a CD8+ depleted SIV-infected rhesus model of neuroAIDS, neuronal injury coincides with viremia and activation of monocyte subsets, as assessed by decreased NAA levels detected by in vivo MRS [100]. Similarly, another study in the same model shows rapid decline in NAA/Cr ratio; decrease in NAA is directly related to neuronal distress. Increases in Cr during disease progression may be related to increased energy demand due to astrocytes and glial activation induced by the entry of infected monocytes into the brain [101]. This dual effect of decreased NAA and increased Cr makes the NAA/Cr ratio a sensitive marker for brain disease status. In addition, the progressive change of NAA and glutamate/glutamine (Glx) in basal ganglia correlated with the CD8+ T cell percentage during the SIV infection [102]. Another study demonstrated that during acute SIV infection a significant decrease in NAA is found in the frontal cortex [103]. 

PET imaging enables the in vivo quantification of microglial activation and neuroinflammation, through radiolabeled ligands that target the translocator protein (TSPO), a naturally expressed receptor on the outer mitochondrial membrane of microglia, macrophages, and astrocytes [104]. During microglial activation, TSPO is significantly upregulated and this can reflect the degree of neuroinflammation. The earliest TSPO radioligand used to assess neuroinflammation in various neurodegenerative diseases was 11C-PK11195, an isoquinoline carboxamide. Earlier work in SIV-infected monkeys using 11C-PK11195 showed evidence of microglial activation in animals with SIVE compared to those without SIVE [105]. In fact, PK11195 binds to infected macrophages and is able to detect longitudinally in SIV-infected macaques, the onset and progression of encephalitis that correlates with areas of synaptic damage in brain tissues [105,106]. More recently, TSPO was imaged in rhesus macaques before and multiple times after inoculation by 18F-DPA714 PET [107]. Using a combination of PET imaging and immunostaining, the authors found evidence of glial (microglia and astrocytes) and neuronal damage in association with very high CSF plasma viral load in SIV-infected monkeys [107]. The use of 18F-fluorodeoxyglucose (FDG)-PET imaging can non-invasively quantify glucose metabolism in the setting of SIV and SHIV infection. In a recent study, brain FDG-PET imaging was used in a group of SIV-infected macaques to longitudinally assess the effect of ART initiation and interruption by monitoring alterations in brain glucose metabolism [108]. The authors observed increased brain glucose metabolism within 1 month of treatment cessation, which may reflect neuroinflammation in the setting of viral rebound, and this is significantly associated with decreased CD4+ and CD8+ T cell counts and increased plasma and CSF viral load [108]. Altogether this evidence suggests that PET imaging may also be considered as biomarkers for SIV disease progression.

**Figure 2:**
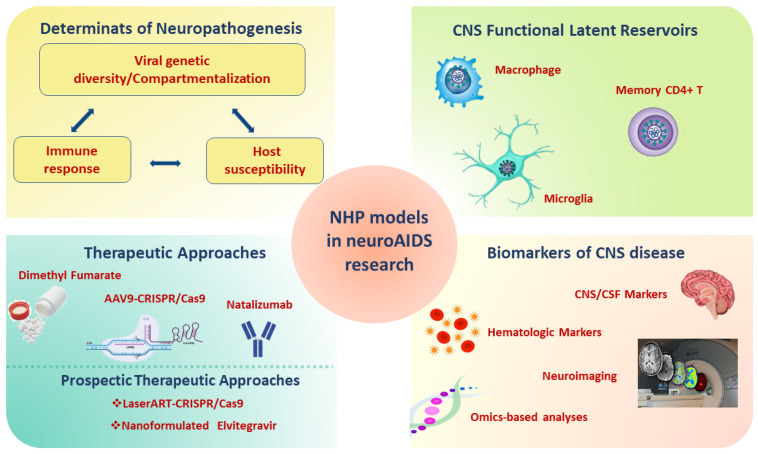
Applications of NHP models infected with SIV/SHIV in neuroAIDS. Abbreviations: SHIV: Simian-Human Immunodeficiency Virus; SIV: Simian Immunodeficiency Virus; NHP: non-human primate; AIDS: Acquired Immunodeficiency Syndrome; ART: antiretroviral therapy; CNS: central nervous system; CSF: cerebrospinal fluid; AAV9: Adeno-Associated Virus 9; CRISP: Clustered Regularly Interspaced Short Palindromic Repeats.

A comprehensive overview of the contribution of the SIV/SHIV monkey models for the study of neuroAIDS is shown in Figure 2.

## 7. Innovative Therapeutic Approaches

Despite ART therapy, secondary complications of the CNS compartment remain at risk in HIV-1-infected individuals [109,110] due to persistent immune activation within the CNS, and to the release of pro-inflammatory cytokines, chemokines, reactive oxygen metabolites, and viral proteins, which ultimately leads to neurodegeneration [110,111]. Oxidative stress and chronic inflammation may contribute to brain injury in acute and chronic SIV infections [111,112]. Thus, protective therapies that aim at suppressing these pathologic pathways would be desirable. In a recent study the ability of dimethyl fumarate (DMF), an antioxidant/anti-inflammatory drug approved for the treatment of multiple sclerosis, has been examined in a rhesus macaque SIV infection model [113,114]. In various animal models, activation of nuclear factor erythroid 2–related factor 2 (Nrf2)/ARE pathway by DMF in the brain is associated with reduced neurodegeneration, and may promote axonal regeneration and block neurotoxicity [115,116]. In a CD8+ T lymphocyte depletion model of SIV neuro-pathogenesis, rhesus macaques received oral DMF prior to SIV infection and through to the necropsy day [114]. Global brain responses to DMF treatment, such as higher brain expression of antioxidant enzymes, lower DNA and protein oxidation, and a more reduced redox state is observed in SIV-infected treated animals as compared to the untreated. This suggests a direct link between enzyme induction and reduced oxidative stress and injury, thus evidencing DMF as a robust antioxidant for the brain [114]. These observations indicate the importance of the macaque SIV infection model, and the potential neuroprotective therapeutic benefit of DMF treatment in persons living with HIV. Another therapeutic approach evaluates the use of the α4-integrin-inhibitor monoclonal antibody natalizumab in SIV-infected rhesus macaques to reduce leukocyte trafficking into the CNS [117]. Natalizumab treatment reduces cell traffic to the brain, the incidence of encephalitis [118] and reduces dorsal root ganglion pathology with decreased inflammation and neuronophagia in SIV-infected macaques [119]. Natalizumab treatment 28 days after infection stabilizes neuronal injury, reduces the number of monocytes/macrophages decreasing productive infection; early treatment at the time of infection blocked monocyte trafficking and SIV RNA is not detectable in the CNS [118]. Blocking trafficking of monocytes/macrophages into the CNS by natalizumab could be an interesting approach to reduce CNS viral load and deleterious interaction of infected monocytes/macrophages with microglia. 

### Future Therapeutic Prospects

Antiretroviral drugs are unable to effectively penetrate the BBB and novel approaches are needed to render more effective drug delivery to viral reservoirs, and eliminate residual latent viral infections. The SIV-infected animal model is helpful to test innovative therapeutic formulations evaluated to date only in vitro or in murine models. For example, nanomedicine has recently been used in the formulation and delivery of drugs that could be a solution to reduce dosages of potentially neurotoxic ART drugs [120]. ART nanoparticles that may effectively cross the BBB, with potentially increased bioavailability without altering neurological integrity, could be an attractive and novel therapeutic solution [120]. A recent study demonstrated that a poloxamer-PLGA nanoformulation loaded with elvitegravir (EVG) is capable of elevating intracellular drug uptake and enhances viral suppression in HIV-1-infected macrophages in vitro, after crossing a BBB mouse model without altering the BBB model integrity, when compared with EVG native drug [121]. Thus, nanoformulated EVG may be an innovative strategy for therapeutic use in CNS drug delivery and should be strengthened in further studies on the SIV-model. 

A recent review has demonstrated the potential use of CRISPR/Cas9 system for HIV-1 therapy [122]. To this aim, SIV-infected monkeys are a promising animal model for the use of CRISPR/Cas9 method to target and edit the SIV genome and for evaluation of proviral SIV elimination. An elegant study provides evidence that inoculation of SIV-infected macaques with Adeno-Associated Virus 9 (AAV9)-CRISPR/Cas9 gene editing construct leads to precise cleavage and removal of fragments of the integrated proviral DNA from the genome of infected blood cells and tissues known to be viral reservoirs, including the brain [123]. The AAV9-CRISPR/Cas9 treatment results in a reduction of proviral DNA in blood and tissues [123]. Thus, the CRISPR/Cas9 system, through the NHP models, might be successfully applied to HIV-1 disease, as it can be used: i) to target proviral DNA for the elimination of provirus; ii) to modify cellular co-factors such as CCR5 which is the main HIV coreceptor; to generate CCR5-mutant (CCR5-delta32) which is associated with protection from, and cure of, HIV infection; and iii) to reactivate host restriction factors during HIV-1 infection, in order to reduce viral infection and clear the provirus. As clinical trials of CRISPR/Cas9 in HIV-1 treatment remains a challenge, the optimization of animal models of HIV-1/AIDS utilizing novel technologies may achieve the clearance and removal of integrated proviral DNA from infected cells and tissues, and may provide high hopes and challenges to translational medical research. Accordingly, a recent study described a sequential long-acting slow-effective release antiviral therapy (LASER ART) and CRISPR-Cas9 therapeutic strategy reservoirs in HIV-1-infected humanized mice [124]. In this study, high hydrophobic lipophilic viral reservoir penetrating antiretroviral prodrugs, coined as long-acting slow-effective release ART, are combined with CRISPR-Cas9-based gene editing technology, providing viral clearance in latent infectious reservoirs in HIV-1-infected humanized mice [124]. New strategies allowed for HIV-1 clearance and elimination of integrated proviral DNA from tissues including the brain, which need validation in further refined non-human primate models and eventually in human clinical studies. 

## Figures and Tables

**Table 1:** Principal SIV/ NHP disease models in neuroHIV research.

Species	Virus	Outcome	Applications	Reference
Rhesus	CD8-depletion/ SIVmac251/239	Rapid disease progression and high incidence of SIVE/ neuroAIDS	neuropathogenesis, viral evolution, ART therapy, omics-based analyses	[11,12,19,20,21]
Rhesus	CD4-depletion/ SIVmac251	Rapid disease progression to neuroAIDS, productive infection in microglia	neuropathogenesis, ART therapy	[22,23]
Pigtailed	SIV/DeltaB670/ SIV/17E-Fr	Rapid disease progression to SIVE/ neuroAIDS	neuropathogenesis, biomarkers of HAND, latent SIV reservoir, therapies for HAND, ART therapy, viral evolution	[24,25]
Rhesus	SIVsm804E	Rapid disease progression and high incidence of SIVE/ neuroAIDS	neuropathogenesis, viral evolution, biomarkers of HAND	[26]
Rhesus	SIVsm804E-CL757	High frequencies of neurological disorders without rapid disease progression, SIVE/neuroAIDS	neuropathogenesis, viral evolution, latent SIV reservoir	[27,28,29]

Abbreviations: SIV: Simian Immunodeficiency Virus;NHP: non-human primate; AIDS: Acquired Immunodeficiency Syndrome; ART: antiretroviral therapy; SIVE: SIV encephalitis; HAND: HIV-associated neurocognitive disorders.

**Table 2:** Biomarkers predicting CNS disease progression.

	Biomarker	Outcome	Reference
**Hematologic**	Plasma viral RNA	Increase	[24]
	Hemoglobin	Decrease	[70]
	Platelet count	Decrease	[70,71]
	Circulating monocytes	No change	[12,70,71]
	Circulating monocytes	Increase	[16]
	Soluble CD163	Increase	[16,70]
**CSF**	CSF viral RNA	Increase	[24]
	CCL2 ^a^	Increase	[32,70]
	IL6	Increase	[70]
	Neopterin	Increase	[70]
	YKL-40 ^b^	Increase	[72,73,74]
	NF-L ^c^	Increase	[70]
	QUIN/TRP ^d^	Increase	[70]
	KP ^e^	Increase	[75,76]
	MicroRNAs ^f^	Upregulation	[70,77,78,79,80,81,82,83,84,85,86,87]
**Omics-based analyses**	Metabolomic ^g^	Increase	[88]
	Proteomic ^h^	Increase	[88,89]
**Neuroimaging**	MRI ^i^	-	[90,91,92,93,94,95,96,97,98,99,100,101,102,103]
	PET ^j^	-	[104,105,106,107,108]

**Abbreviations: ^a^:** monocyte chemoattractant protein 1 (MCP-1) or CCL2; **^b^:** human cartilage glycoprotein 39; **^c^:** neurofilament light chain; **^d^:** quinolinic acid/tryptophan ratio; **^e^:** kynurenine pathway; **^f^:** miR-125b, miR-146a, miR-21, miR-142-5p, miR-1423p; analysis of: **^g^:** phospholipids, free fatty acids, phospholipases; **^h^:** alpha-1-antitrypsin, complement C3, hemopexin, IgM heavy chain, plasminogen; **^i^:** multiple magnetic resonance imaging (MRI), **^j^:** positron emission tomography (PET), cerebrospinal fluid (CSF).

## References

[1] Balcom E.F., Roda W.C., Cohen E.A., Li M.Y., Power C. (2019). HIV-1 persistence in the central nervous system: Viral and host determinants during antiretroviral therapy. Curr. Opin. Virol..

[2] Martinez-Navio J.M. (2021). Neurological complications during HIV infection. Explor. Neuroprotective Ther..

[3] Cafaro A., Caputo A., Fracasso C., Maggiorella M.T., Goletti D., Baroncelli S., Pace M., Sernicola L., Koanga-Mogtomo M.L., Betti M. (1999). Control of SHIV-89.6P-infection of cynomolgus monkeys by HIV-1 Tat protein vaccine. Nat. Med..

[4] Liang B., Li H., Li L., Omange R.W., Hai Y., Luo M. (2019). Current advances in HIV vaccine preclinical studies using Macaque models. Vaccine.

[5] Thippeshappa R., Kimata J.T., Kaushal D. (2020). Toward a macaque model of HIV-1 infection: Roadblocks, progress, and future strategies. Front. Microbiol..

[6] Williams K., Lackner A., Mallard J. (2016). Non-human primate models of SIV infection and CNS neuropathology. Curr. Opin. Virol..

[7] Beck S.E., Queen S.E., Metcalf Pate K.A., Mangus L.M., Abreu C.M., Gama L., Witwer K.W., Adams R.J., Zink M.C., Clements J.E. (2018). An SIV/macaque model targeted to study HIV-associated neurocognitive disorders. J. Neurovirol..

[8] Mallard J., Williams K.C. (2018). Animal models of HIV-associated disease of the central nervous system. Handb. Clin. Neurol..

[9] Milush J.M., Chen H.L., Atteberry G., Sodora D.L. (2013). Early detection of simian immunodeficiency virus in the central nervous system following oral administration to rhesus macaques. Front. Immunol..

[10] Barouch D.H., Ghneim K., Bosche W.J., Li Y., Berkemeier B., Hull M., Bhattacharyya S., Cameron M., Liu J., Smith K. (2016). Rapid inflammasome activation following mucosal SIV infection of rhesus monkeys. Cell.

[11] Annamalai L., Bhaskar V., Pauley D.R., Knight H., Williams K., Lentz M., Ratai E., Westmoreland S.V., González R.G., O’Neil S.P. (2010). Impact of short-term combined antiretroviral therapy on brain virus burden in simian immunodeficiency virus-infected and CD8+ lymphocyte-depleted rhesus macaques. Am. J. Pathol..

[12] Bissel S.J., Wang G., Trichel A.M., Murphey-Corb M., Wiley C.A. (2006). Longitudinal analysis of monocyte/macrophage infection in simian immunodeficiency virus-infected, CD8+ T-cell-depleted macaques that develop lentiviral encephalitis. Am. J. Pathol..

[13] Williams K.C., Corey S., Westmoreland S.V., Pauley D., Knight H., de Bakker C., Alvarez X., Lackner A.A. (2001). Perivascular macrophages are the primary cell type productively infected by simian immunodeficiency virus in the brains of macaques: Implications for the neuropathogenesis of AIDS. J. Exp. Med..

[14] Gama L., Abreu C., Shirk E.N., Queen S.E., Beck S.E., Metcalf Pate K.A., Bullock B.T., Zink M.C., Mankowski J.L., Clements J.E. (2018). SIV latency in macrophages in the CNS. Curr. Top. Microbiol. Immunol..

[15] Ancuta P., Kamat A., Kunstman K.J., Kim E.Y., Autissier P., Wurcel A., Zaman T., Stone D., Mefford M., Morgello S. (2008). Microbial translocation is associated with increased monocyte activation and dementia in AIDS patients. PLoS ONE.

[16] Burdo T.H., Soulas C., Orzechowski K., Button J., Krishnan A., Sugimoto C., Alvarez X., Kuroda M.J., Williams K.C. (2010). Increased monocyte turnover from bone marrow correlates with severity of SIV encephalitis and CD163 levels in plasma. PLoS Pathog..

[17] Nowlin B.T., Burdo T.H., Midkiff C.C., Salemi M., Alvarez X., Williams K.C. (2015). SIV encephalitis lesions are composed of CD163(+) macrophages present in the central nervous system during early SIV infection and SIV-positive macrophages recruited terminally with AIDS. Am. J. Pathol..

[18] Filipowicz A.R., McGary C.M., Holder G.E., Lindgren A.A., Johnson E.M., Sugimoto C., Kuroda M.J., Kim W.K. (2016). Proliferation of perivascular macrophages contributes to the development of encephalitic lesions in HIV-infected humans and in SIV-infected macaques. Sci. Rep..

[19] Strickland S.L., Rife B.D., Lamers S.L., Nolan D.J., Veras N., Prosperi M., Burdo T.H., Autissier P., Nowlin B., Goodenow M.M. (2014). Spatiotemporal dynamics of simian immunodeficiency virus brain infection in CD8+ lymphocyte-depleted rhesus macaques with neuroAIDS. J. Gen. Virol..

[20] Marcondes M.C., Morsey B., Emanuel K., Lamberty B.G., Flynn C.T., Fox H.S. (2015). CD8+ T cells maintain suppression of Simian Immunodeficiency Virus in the central nervous system. J. Infect. Dis..

[21] Rife Magalis B., Nolan D.J., Autissier P., Burdo T.H., Williams K.C., Salemi M. (2017). Insights into the impact of CD8+ immune modulation on Human Immunodeficiency Virus evolutionary dynamics in distinct anatomical compartments by using Simian Immunodeficiency Virus-infected macaque models of AIDS progression. J. Virol..

[22] Ortiz A.M., Klatt N.R., Li B., Yi Y., Tabb B., Hao X.P., Sternberg L., Lawson B., Carnathan P.M., Cramer E. (2011). Depletion of CD4⁺ T cells abrogates post-peak decline of viremia in SIV-infected rhesus macaques. J. Clin. Investig..

[23] Micci L., Alvarez X., Iriele R.I., Ortiz A.M., Ryan E.S., McGary C.S., Deleage C., McAtee B.B., He T., Apetrei C. (2014). CD4 depletion in SIV-infected macaques results in macrophage and microglia infection with rapid turnover of infected cells. PLoS Pathog..

[24] Zink M.C., Suryanarayana K., Mankowski J.L., Shen A., Piatak M., Spelman J.P., Carter D.L., Adams R.J., Lifson J.D., Clements J.E. (1999). High viral load in the cerebrospinal fluid and brain correlates with severity of simian immunodeficiency virus encephalitis. J. Virol..

[25] Dorsey J.L., Mangus L.M., Hauer P., Ebenezer G.J., Queen S.E., Laast V.A., Adams R.J., Mankowski J.L. (2015). Persistent peripheral nervous system damage in Simian Immunodeficiency Virus-infected macaques receiving antiretroviral therapy. J. Neuropathol. Exp. Neurol..

[26] Hirsch V., Adger-Johnson D., Campbell B., Goldstein S., Brown C., Elkins W.R., Montefiori D.C. (1997). A molecularly cloned, pathogenic, neutralization-resistant simian immunodeficiency virus, SIVsmE543-3. J. Virol..

[27] Matsuda K., Riddick N.E., Lee C.A., Puryear S.B., Wu F., Lafont B., Whitted S., Hirsch V.M. (2017). A SIV molecular clone that targets the CNS and induces neuroAIDS in rhesus macaques. PLoS Pathog..

[28] Lee C.A., Beasley E., Sundar K., Smelkinson M., Vinton C., Deleage C., Matsuda K., Wu F., Estes J.D., Lafont B. (2020). Simian Immunodeficiency Virus-infected memory CD4^+^ T cells infiltrate to the site of infected macrophages in the neuroparenchyma of a chronic macaque model of neurological complications of AIDS. mBio.

[29] Hsu D.C., Sunyakumthorn P., Wegner M., Schuetz A., Silsorn D., Estes J.D., Deleage C., Tomusange K., Lakhashe S.K., Ruprecht R.M. (2018). Central nervous system inflammation and infection during early, nonaccelerated Simian-Human Immunodeficiency Virus infection in rhesus macaques. J. Virol..

[30] Shytaj I.L., Norelli S., Chirullo B., Della Corte A., Collins M., Yalley-Ogunro J., Greenhouse J., Iraci N., Acosta E.P., Barreca M.L. (2012). A highly intensified ART regimen induces long-term viral suppression and restriction of the viral reservoir in a simian AIDS model. PLoS Pathog..

[31] Clements J.E., Li M., Gama L., Bullock B., Carruth L.M., Mankowski J.L., Zink M.C. (2005). The central nervous system is a viral reservoir in simian immunodeficiency virus-infected macaques on combined antiretroviral therapy: A model for human immunodeficiency virus patients on highly active antiretroviral therapy. J. Neurovirol..

[32] Zink M.C., Brice A.K., Kelly K.M., Queen S.E., Gama L., Li M., Adams R.J., Bartizal C., Varrone J., Rabi S. (2010). Simian Immunodeficiency Virus-infected macaques treated with highly active antiretroviral therapy have reduced central nervous system viral replication and inflammation but persistence of viral DNA. J. Infect. Dis..

[33] Graham D.R., Gama L., Queen S.E., Li M., Brice A.K., Kelly K.M., Mankowski J.L., Clements J.E., Zink M.C. (2011). Initiation of HAART during acute simian immunodeficiency virus infection rapidly controls virus replication in the CNS by enhancing immune activity and preserving protective immune responses. J. Neurovirol..

[34] Estes J.D., Kityo C., Ssali F., Swainson L., Makamdop K.N., Del Prete G.Q., Deeks S.G., Luciw P.A., Chipman J.G., Beilman G.J. (2017). Defining total-body AIDS-virus burden with implications for curative strategies. Nat. Med..

[35] Perez S., Johnson A.M., Xiang S.H., Li J., Foley B.T., Doyle-Meyers L., Panganiban A., Kaur A., Veazey R.S., Wu Y. (2018). Persistence of SIV in the brain of SIV-infected chinese rhesus macaques with or without antiretroviral therapy. J. Neurovirol..

[36] Mavigner M., Habib J., Deleage C., Rosen E., Mattingly C., Bricker K., Kashuba A., Amblard F., Schinazi R.F., Lawson B. (2018). Simian Immunodeficiency Virus persistence in cellular and anatomic reservoirs in antiretroviral therapy-suppressed infant rhesus macaques. J. Virol..

[37] Avalos C.R., Price S.L., Forsyth E.R., Pin J.N., Shirk E.N., Bullock B.T., Queen S.E., Li M., Gellerup D., O’Connor S.L. (2016). Quantitation of productively infected monocytes and macrophages of Simian Immunodeficiency Virus-infected macaques. J. Virol..

[38] Avalos C.R., Abreu C.M., Queen S.E., Li M., Price S., Shirk E.N., Engle E.L., Forsyth E., Bullock B.T., Mac Gabhann F. (2017). Brain macrophages in Simian Immunodeficiency Virus-infected, antiretroviral-suppressed macaques: A functional latent reservoir. mBio.

[39] Abreu C., Shirk E.N., Queen S.E., Beck S.E., Mangus L.M., Pate K., Mankowski J.L., Gama L., Clements J.E. (2019). Brain macrophages harbor latent, infectious simian immunodeficiency virus. AIDS.

[40] Abreu C., Shirk E.N., Queen S.E., Mankowski J.L., Gama L., Clements J.E. (2019). A quantitative approach to SIV functional latency in brain macrophages. J. Neuroimmune Pharmacol..

[41] Reynoso R., Wieser M., Ojeda D., Bönisch M., Kühnel H., Bolcic F., Quendler H., Grillari J., Grillari-Voglauer R., Quarleri J. (2012). HIV-1 induces telomerase activity in monocyte-derived macrophages, possibly safeguarding one of its reservoirs. J. Virol..

[42] Gama L., Abreu C.M., Shirk E.N., Price S.L., Li M., Laird G.M., Pate K.A., Wietgrefe S.W., O’Connor S.L., Pianowski L. (2017). Reactivation of simian immunodeficiency virus reservoirs in the brain of virally suppressed macaques. AIDS.

[43] Jenkins S.J., Ruckerl D., Cook P.C., Jones L.H., Finkelman F.D., van Rooijen N., MacDonald A.S., Allen J.E. (2011). Local macrophage proliferation, rather than recruitment from the blood, is a signature of TH2 inflammation. Science.

[44] Salemi M., Rife B. (2016). Phylogenetics and Phyloanatomy of HIV/SIV intra-host compartments and reservoirs: The key role of the central nervous system. Curr. HIV Res..

[45] Bednar M.M., Sturdevant C.B., Tompkins L.A., Arrildt K.T., Dukhovlinova E., Kincer L.P., Swanstrom R. (2015). Compartmentalization, viral evolution, and viral latency of HIV in the CNS. Curr. HIV/AIDS Rep..

[46] Schnell G., Spudich S., Harrington P., Price R.W., Swanstrom R. (2009). Compartmentalized human immunodeficiency virus type 1 originates from long-lived cells in some subjects with HIV-1-associated dementia. PLoS Pathog..

[47] Schnell G., Joseph S., Spudich S., Price R.W., Swanstrom R. (2011). HIV-1 replication in the central nervous system occurs in two distinct cell types. PLoS Pathog..

[48] Yen P.J., Mefford M.E., Hoxie J.A., Williams K.C., Desrosiers R.C., Gabuzda D. (2014). Identification and characterization of a macrophage-tropic SIV envelope glycoprotein variant in blood from early infection in SIVmac251-infected macaques. Virology.

[49] Beck S.E., Queen S.E., Viscidi R., Johnson D., Kent S.J., Adams R.J., Tarwater P.M., Mankowski J.L. (2016). Central nervous system-specific consequences of simian immunodeficiency virus Gag escape from major histocompatibility complex class I-mediated control. J. Neurovirol..

[50] Queen S.E., Mears B.M., Kelly K.M., Dorsey J.L., Liao Z., Dinoso J.B., Gama L., Adams R.J., Zink M.C., Clements J.E. (2011). Replication-competent simian immunodeficiency virus (SIV) Gag escape mutations archived in latent reservoirs during antiretroviral treatment of SIV-infected macaques. J. Virol..

[51] Rife B.D., Nolan D.J., Lamers S.L., Autissier P., Burdo T., Williams K.C., Salemi M. (2016). Evolution of neuroadaptation in the periphery and purifying selection in the brain contribute to compartmentalization of Simian Immunodeficiency Virus (SIV) in the brains of rhesus macaques with SIV-associated encephalitis. J. Virol..

[52] Lamers S.L., Nolan D.J., Rife B.D., Fogel G.B., McGrath M.S., Burdo T.H., Autissier P., Williams K.C., Goodenow M.M., Salemi M. (2015). Tracking the Emergence of Host-Specific Simian Immunodeficiency Virus env and nef populations reveals nef early adaptation and convergent evolution in brain of naturally progressing rhesus macaques. J. Virol..

[53] Capone A., Lo Presti A., Sernicola L., Farcomeni S., Ferrantelli F., Maggiorella M.T., Mee E.T., Rose N.J., Cella E., Ciccozzi M. (2018). Genetic diversity in the env V1-V2 region of proviral quasispecies from long-term controller MHC-typed cynomolgus macaques infected with SHIVSF162P4cy. J. Gen. Virol..

[54] Mühl T., Krawczak M., Ten Haaft P., Hunsmann G., Sauermann U. (2002). MHC class I alleles influence set-point viral load and survival time in simian immunodeficiency virus-infected rhesus monkeys. J. Immunol..

[55] O’Connor D.H., Mothe B.R., Weinfurter J.T., Fuenger S., Rehrauer W.M., Jing P., Rudersdorf R.R., Liebl M.E., Krebs K., Vasquez J. (2003). Major histocompatibility complex class I alleles associated with slow simian immunodeficiency virus disease progression bind epitopes recognized by dominant acute-phase cytotoxic-T-lymphocyte responses. J. Virol..

[56] Borsetti A., Maggiorella M.T., Sernicola L., Bellino S., Ferrantelli F., Belli R., Fulgenzi D., Mee E.T., Rose N.J., Cafaro A. (2012). Influence of MHC class I and II haplotypes on the experimental infection of Mauritian cynomolgus macaques with SHIVSF162P4cy. Tissue Antigens.

[57] Borsetti A., Ferrantelli F., Maggiorella M.T., Sernicola L., Bellino S., Gallinaro A., Farcomeni S., Mee E.T., Rose N.J., Cafaro A. (2014). Effect of MHC haplotype on immune response upon experimental SHIVSF162P4cy infection of Mauritian cynomolgus macaques. PLoS ONE.

[58] Yant L.J., Friedrich T.C., Johnson R.C., May G.E., Maness N.J., Enz A.M., Lifson J.D., O’Connor D.H., Carrington M., Watkins D.I. (2006). The high-frequency major histocompatibility complex class I allele Mamu-B*17 is associated with control of simian immunodeficiency virus SIVmac239 replication. J. Virol..

[59] Mankowski J.L., Queen S.E., Fernandez C.S., Tarwater P.M., Karper J.M., Adams R.J., Kent S.J. (2008). Natural host genetic resistance to lentiviral CNS disease: A neuroprotective MHC class I allele in SIV-infected macaques. PLoS ONE.

[60] Deng K., Pertea M., Ronvaux A., Wang L., Durand C.M., Ghiaur G., Lai J., McHugh H.L., Hao H., Zhang H. (2015). Broad CTL response is required to clear latent HIV-1 due to dominance of escape mutations. Nature.

[61] Matsuda K., Dang Q., Brown C.R., Keele B.F., Wu F., Ourmanov I., Goeken R., Whitted S., Riddick N.E., Buckler-White A. (2014). Characterization of simian immunodeficiency virus (SIV) that induces SIV encephalitis in rhesus macaques with high frequency: Role of TRIM5 and major histocompatibility complex genotypes and early entry to the brain. J. Virol..

[62] Ajasin D., Eugenin E.A. (2020). HIV-1 Tat: Role in bystander toxicity. Front. Cell. Infect. Microbiol..

[63] Yarandi S.S., Duggan M.R., Sariyer I.K. (2021). Emerging role of nef in the development of HIV associated neurological disorders. J. Neuroimmune Pharmacol..

[64] Thompson K.A., Kent S.J., Gahan M.E., Purcell D.F., McLean C.A., Preiss S., Dale C.J., Wesselingh S.L. (2003). Decreased neurotropism of nef long terminal repeat (nef/LTR)-deleted simian immunodeficiency virus. J. Neurovirol..

[65] Lehmann M.H., Lehmann J.M., Erfle V. (2019). Nef-induced CCL2 expression contributes to HIV/SIV brain invasion and neuronal dysfunction. Front. Immunol..

[66] Yarandi S.S., Robinson J.A., Vakili S., Donadoni M., Burdo T.H., Sariyer I.K. (2020). Characterization of Nef expression in different brain regions of SIV-infected macaques. PLoS ONE.

[67] Wang J., Zhang Y., Xu Q., Qiu J., Zheng H., Ye X., Xue Y., Yin Y., Zhang Z., Liu Y. (2017). Menin mediates Tat-induced neuronal apoptosis in brain frontal cortex of SIV-infected macaques and in Tat-treated cells. Oncotarget.

[68] Chivero E.T., Guo M.L., Periyasamy P., Liao K., Callen S.E., Buch S. (2017). HIV-1 Tat primes and activates microglial NLRP3 inflammasome-mediated neuroinflammation. J. Neurosci..

[69] Sil S., Hu G., Liao K., Niu F., Callen S., Periyasamy P., Fox H.S., Buch S. (2020). HIV-1 Tat-mediated astrocytic amyloidosis involves the HIF-1α/lncRNA BACE1-AS axis. PLoS Biol..

[70] Beck S.E., Queen S.E., Witwer K.W., Metcalf Pate K.A., Mangus L.M., Gama L., Adams R.J., Clements J.E., Zink C.M., Mankowski J.L. (2015). Paving the path to HIV neurotherapy: Predicting SIV CNS disease. Eur. J. Pharmacol..

[71] Wachtman L.M., Tarwater P.M., Queen S.E., Adams R.J., Mankowski J.L. (2006). Platelet decline: An early predictive hematologic marker of simian immunodeficiency virus central nervous system disease. J. Neurovirol..

[72] Kolson D.L. (2008). YKL-40: A candidate biomarker for simian immunodeficiency virus and human immunodeficiency virus encephalitis?. Am. J. Pathol..

[73] Bonneh-Barkay D., Bissel S.J., Wang G., Fish K.N., Nicholl G.C., Darko S.W., Medina-Flores R., Murphey-Corb M., Rajakumar P.A., Nyaundi J. (2008). YKL-40, a marker of simian immunodeficiency virus encephalitis, modulates the biological activity of basic fibroblast growth factor. Am. J. Pathol..

[74] Bissel S.J., Kofler J., Nyaundi J., Murphey-Corb M., Wisniewski S.R., Wiley C.A. (2016). Cerebrospinal fluid biomarkers of Simian Immunodeficiency Virus encephalitis: CSF biomarkers of SIV encephalitis. J. Neuroimmune Pharmacol..

[75] Drewes J.L., Meulendyke K.A., Liao Z., Witwer K.W., Gama L., Ubaida-Mohien C., Li M., Notarangelo F.M., Tarwater P.M., Schwarcz R. (2015). Quinolinic acid/tryptophan ratios predict neurological disease in SIV-infected macaques and remain elevated in the brain under cART. J. Neurovirol..

[76] Tavano B., Tsipouri V., Hardy G., Royle C.M., Keegan M.R., Fuchs D., Patterson S., Almond N., Berry N., Ham C. (2017). Immune responses in the central nervous system are anatomically segregated in a non-human primate model of human immunodeficiency virus infection. Front. Immunol..

[77] Reynoso R., Laufer N., Hackl M., Skalicky S., Monteforte R., Turk G., Carobene M., Quarleri J., Cahn P., Werner R. (2014). MicroRNAs differentially present in the plasma of HIV elite controllers reduce HIV infection in vitro. Sci. Rep..

[78] Witwer K.W., Watson A.K., Blankson J.N., Clements J.E. (2012). Relationships of PBMC microRNA expression, plasma viral load, and CD4+ T-cell count in HIV-1-infected elite suppressors and viremic patients. Retrovirology.

[79] Noorbakhsh F., Ramachandran R., Barsby N., Ellestad K.K., LeBlanc A., Dickie P., Baker G., Hollenberg M.D., Cohen E.A., Power C. (2010). MicroRNA profiling reveals new aspects of HIV neurodegeneration: Caspase-6 regulates astrocyte survival. FASEB J..

[80] Rom S., Rom I., Passiatore G., Pacifici M., Radhakrishnan S., Del Valle L., Piña-Oviedo S., Khalili K., Eletto D., Peruzzi F. (2010). CCL8/MCP-2 is a target for mir-146a in HIV-1-infected human microglial cells. FASEB J..

[81] Sisk J.M., Witwer K.W., Tarwater P.M., Clements J.E. (2013). SIV replication is directly downregulated by four antiviral miRNAs. Retrovirology.

[82] Yelamanchili S.V., Fox H.S. (2010). Defining larger roles for “tiny” RNA molecules: Role of miRNAs in neurodegeneration research. J. Neuroimmune Pharmacol..

[83] Yelamanchili S.V., Lamberty B.G., Rennard D.A., Morsey B.M., Hochfelder C.G., Meays B.M., Levy E., Fox H.S. (2015). MiR-21 in extracellular vesicles leads to neurotoxicity via TLR7 signaling in SIV neurological disease. PLoS Pathog..

[84] Moens U. (2009). Silencing viral microRNA as a novel antiviral therapy?. J. Biomed. Biotechnol..

[85] Swaminathan S., Murray D.D., Kelleher A.D. (2012). The role of microRNAs in HIV-1 pathogenesis and therapy. AIDS.

[86] Want E.J., Nordström A., Morita H., Siuzdak G. (2007). From exogenous to endogenous: The inevitable imprint of mass spectrometry in metabolomics. J. Prot. Res..

[87] Lane T.E., Buchmeier M.J., Watry D.D., Fox H.S. (1996). Expression of inflammatory cytokines and inducible nitric oxide synthase in brains of SIV-infected rhesus monkeys: Applications to HIV-induced central nervous system disease. Mol. Med..

[88] Wikoff W.R., Pendyala G., Siuzdak G., Fox H.S. (2008). Metabolomic analysis of the cerebrospinal fluid reveals changes in phospholipase expression in the CNS of SIV-infected macaques. J. Clin. Investig..

[89] Pendyala G., Trauger S.A., Kalisiak E., Ellis R.J., Siuzdak G., Fox H.S. (2009). Cerebrospinal fluid proteomics reveals potential pathogenic changes in the brains of SIV-infected monkeys. J. Prot. Res..

[90] Winkler J.M., Chaudhuri A.D., Fox H.S. (2012). Translating the brain transcriptome in neuroAIDS: From non-human primates to humans. J. Neuroimmune Pharmacol..

[91] Roberts E.S., Burudi E.M., Flynn C., Madden L.J., Roinick K.L., Watry D.D., Zandonatti M.A., Taffe M.A., Fox H.S. (2004). Acute SIV infection of the brain leads to upregulation of IL6 and interferon-regulated genes: Expression patterns throughout disease progression and impact on neuroAIDS. J. Neuroimmunol..

[92] Gersten M., Alirezaei M., Marcondes M.C., Flynn C., Ravasi T., Ideker T., Fox H.S. (2009). An integrated systems analysis implicates EGR1 downregulation in simian immunodeficiency virus encephalitis-induced neural dysfunction. J. Neurosci..

[93] Mavian C., Ramirez-Mata A.S., Dollar J.J., Nolan D.J., Cash M., White K., Rich S.N., Magalis B.R., Marini S., Prosperi M. (2021). Brain tissue transcriptomic analysis of SIV-infected macaques identifies several altered metabolic pathways linked to neuropathogenesis and poly (ADP-ribose) polymerases (PARPs) as potential therapeutic targets. J. Neurovirol..

[94] Li C., Zhang X., Komery A., Li Y., Novembre F.J., Herndon J.G. (2011). Longitudinal diffusion tensor imaging and perfusion MRI investigation in a macaque model of neuro-AIDS: A preliminary study. NeuroImage.

[95] Zhao J., Jing B., Chen F., Liu J., Wang Y., Li H. (2017). Altered regional homogeneity of brain spontaneous signals in SIV infected rhesus macaque model. Magn. Reson. Imaging.

[96] Zhao J., Chen F., Ren M., Li L., Li A., Jing B., Li H. (2019). Low-frequency fluctuation characteristics in rhesus macaques with SIV infection: A resting-state fMRI study. J. Neurovirol..

[97] Li C.X., Herndon J.G., Novembre F.J., Zhang X. (2015). A longitudinal magnetization transfer imaging evaluation of brain injury in a macaque model of neuroAIDS. AIDS Res. Hum. Retrovir..

[98] Greco J.B., Westmoreland S.V., Ratai E.M., Lentz M.R., Sakaie K., He J., Sehgal P.K., Masliah E., Lackner A.A., González R.G. (2004). In vivo 1H MRS of brain injury and repair during acute SIV infection in the macaque model of neuroAIDS. Magn. Reson. Med..

[99] Fuller R.A., Westmoreland S.V., Ratai E., Greco J.B., Kim J.P., Lentz M.R., He J., Sehgal P.K., Masliah E., Halpern E. (2004). A prospective longitudinal in vivo 1H MR spectroscopy study of the SIV/macaque model of neuroAIDS. BMC Neurosci..

[100] Williams K., Westmoreland S., Greco J., Ratai E., Lentz M., Kim W.K., Fuller R.A., Kim J.P., Autissier P., Sehgal P.K. (2005). Magnetic resonance spectroscopy reveals that activated monocytes contribute to neuronal injury in SIV neuroAIDS. J. Clin. Investig..

[101] Ratai E.M., Annamalai L., Burdo T., Joo C.G., Bombardier J.P., Fell R., Hakimelahi R., He J., Lentz M.R., Campbell J. (2011). Brain creatine elevation and N-Acetylaspartate reduction indicates neuronal dysfunction in the setting of enhanced glial energy metabolism in a macaque model of neuroAIDS. Magn. Reson. Med..

[102] Li C.X., Zhang X., Komery A., Li Y., Mao H., Herndon J.G., Novembre F.J. (2014). Longitudinal cerebral metabolic changes in pig-tailed macaques infected with the neurovirulent virus SIVsmmFGb. J. Neurovirol..

[103] Ratai E.M., Pilkenton S.J., Greco J.B., Lentz M.R., Bombardier J.P., Turk K.W., He J., Joo C.G., Lee V., Westmoreland S. (2009). In vivo proton magnetic resonance spectroscopy reveals region specific metabolic responses to SIV infection in the macaque brain. BMC Neurosci..

[104] Scharko A.M., Perlman S.B., Hinds P.W., Hanson J.M., Uno H., Pauza C.D. (1996). Whole body positron emission tomography imaging of simian immunodeficiency virus-infected rhesus macaques. Proc. Natl. Acad. Sci. USA.

[105] Venneti S., Lopresti B.J., Wang G., Bissel S.J., Mathis C.A., Meltzer C.C., Boada F., Capuano S., Kress G.J., Davis D.K. (2004). PET imaging of brain macrophages using the peripheral benzodiazepine receptor in a macaque model of neuroAIDS. J. Clin. Investig..

[106] Venneti S., Bonneh-Barkay D., Lopresti B.J., Bissel S.J., Wang G., Mathis C.A., Piatak M., Lifson J.D., Nyaundi J.O., Murphey-Corb M. (2008). Longitudinal in vivo positron emission tomography imaging of infected and activated brain macrophages in a macaque model of human immunodeficiency virus encephalitis correlates with central and peripheral markers of encephalitis and areas of synaptic degeneration. Am. J. Pathol..

[107] Hammoud D.A., Sinharay S., Shah S., Schreiber-Stainthorp W., Maric D., Muthusamy S., Lee D.E., Lee C.A., Basuli F., Reid W.C. (2019). Neuroinflammatory changes in relation to cerebrospinal fluid viral load in Simian Immunodeficiency Virus encephalitis. mBio.

[108] Sinharay S., Hammoud D.A. (2019). Brain PET Imaging: Value for understanding the pathophysiology of HIV-associated Neurocognitive Disorder (HAND). Curr. HIV/AIDS Rep..

[109] Gill A.J., Kolson D.L. (2014). Chronic inflammation and the role for cofactors (hepatitis C, drug abuse, antiretroviral drug toxicity, aging) in HAND persistence. Curr. HIV/AIDS Rep..

[110] McGuire J.L., Gill A.J., Douglas S.D., Kolson D.L., CNS HIV Anti-Retroviral Therapy Effects Research (CHARTER) Group (2015). Central and peripheral markers of neurodegeneration and monocyte activation in HIV-associated neurocognitive disorders. J. Neurovirol..

[111] de Almeida S.M., Rotta I., Ribeiro C.E., Smith D., Wang R., Judicello J., Potter M., Vaida F., Letendre S., Ellis R.J. (2016). Blood-CSF barrier and compartmentalization of CNS cellular immune response in HIV infection. J. Neuroimmunol..

[112] Garcia-Mesa Y., Garza R., Diaz Ortiz M.E., Gruenewald A.L., Bastien B.L., Lobrovich R., Irwin D.J., Betts M.R., Silvestri G., Kolson D.L. (2020). Regional brain recovery from acute synaptic injury in Simian Immunodeficiency Virus-infected rhesus macaques associates with heme oxygenase isoform expression. J. Virol..

[113] Cross S.A., Cook D.R., Chi A.W., Vance P.J., Kolson L.L., Wong B.J., Jordan-Sciutto K.L., Kolson D.L. (2011). Dimethyl fumarate, an immune modulator and inducer of the antioxidant response, suppresses HIV replication and macrophage-mediated neurotoxicity: A novel candidate for HIV neuroprotection. J. Immunol..

[114] Garcia-Mesa Y., Xu H.N., Vance P., Gruenewald A.L., Garza R., Midkiff C., Alvarez-Hernandez X., Irwin D.J., Gill A.J., Kolson D.L. (2021). Dimethyl fumarate, an approved multiple sclerosis treatment, reduces brain oxidative stress in SIV-infected rhesus macaques: Potential therapeutic repurposing for HIV neuroprotection. Antioxidants.

[115] Iniaghe L.O., Krafft P.R., Klebe D.W., Omogbai E., Zhang J.H., Tang J. (2015). Dimethyl fumarate confers neuroprotection by casein kinase 2 phosphorylation of Nrf2 in murine intracerebral hemorrhage. Neurobiol. Dis..

[116] Jing X., Shi H., Zhang C., Ren M., Han M., Wei X., Zhang X., Lou H. (2015). Dimethyl fumarate attenuates 6-OHDA-induced neurotoxicity in SH-SY5Y cells and in animal model of Parkinson’s disease by enhancing Nrf2 activity. Neuroscience.

[117] Ambrosius B., Gold R., Chan A., Faissner S. (2019). Antineuroinflammatory drugs in HIV-associated neurocognitive disorders as potential therapy. Neurol. Neuroimmunol. Neuroinflamm..

[118] Campbell J.H., Ratai E.M., Autissier P., Nolan D.J., Tse S., Miller A.D., González R.G., Salemi M., Burdo T.H., Williams K.C. (2014). Anti-α4 antibody treatment blocks virus traffic to the brain and gut early, and stabilizes CNS injury late in infection. PLoS Pathog..

[119] Lakritz J.R., Thibault D.M., Robinson J.A., Campbell J.H., Miller A.D., Williams K.C., Burdo T.H. (2016). α4-integrin antibody treatment blocks monocyte/macrophage traffic to, vascular cell adhesion molecule-1 expression in, and pathology of the dorsal root ganglia in an SIV macaque model of HIV-peripheral neuropathy. Am. J. Pathol..

[120] Osborne O., Peyravian N., Nair M., Daunert S., Toborek M. (2020). The paradox of HIV blood-brain barrier penetrance and antiretroviral drug delivery deficiencies. Trends Neurosci..

[121] Gong Y., Chowdhury P., Nagesh P., Rahman M.A., Zhi K., Yallapu M.M., Kumar S. (2020). Novel elvitegravir nanoformulation for drug delivery across the blood-brain barrier to achieve HIV-1 suppression in the CNS macrophages. Sci. Rep..

[122] Xiao Q., Guo D., Chen S. (2019). Application of CRISPR/Cas9-Based Gene Editing in HIV-1/AIDS Therapy. Front. Cell. Infect. Microbiol..

[123] Mancuso P., Chen C., Kaminski R., Gordon J., Liao S., Robinson J.A., Smith M.D., Liu H., Sariyer I.K., Sariyer R. (2020). CRISPR based editing of SIV proviral DNA in ART treated non-human primates. Nat. Commun..

[124] Dash P.K., Kaminski R., Bella R., Su H., Mathews S., Ahooyi T.M., Chen C., Mancuso P., Sariyer R., Ferrante P. (2019). Sequential LASER ART and CRISPR treatments eliminate HIV-1 in a subset of infected humanized mice. Nat. Commun..

